# Heterozygous X-linked Alport syndrome in a pregnant woman: A case report

**DOI:** 10.1177/2050313X261429614

**Published:** 2026-03-04

**Authors:** Caroline Gee, Matthew D. Nguyen, Dao Le, Ramy Hanna

**Affiliations:** 1 Division of Nephrology, Hypertension, and Kidney Transplantation, Department of Medicine, University of California, Irvine, USA; 2 Department of Medicine, University of Southern California/Los Angeles General Medical Center, USA

**Keywords:** X-linked Alport syndrome, pregnancy, *COL4A5*, proteinuria, genetic counseling, case report

## Abstract

Alport syndrome is a genetic disorder of chronic kidney disease, hearing loss, and ocular abnormalities, caused by mutations in type IV collagen. While X-linked Alport Syndrome demonstrates characteristic severe renal failure in males, it has a variable presentation in females. Here, we present a case of a pregnant woman who was found to have X-linked Alport Syndrome with a heterozygous c.3587G>A (p.Gly1196Glu) mutation in the *COL4A5* gene. The patient presented in her third trimester of pregnancy with preexisting chronic proteinuria. Family history was notable for kidney failure in her mother and two brothers, consistent with suspected hereditary nephropathy. She developed worsening proteinuria and new-onset hematuria, without preeclampsia or renal failure, and was induced at 39 weeks with an uncomplicated delivery. Genetic testing revealed a heterozygous c.3587G>A (p.Gly1196Glu) mutation in the *COL4A5* gene, consistent with X-linked Alport Syndrome. Postpartum, the patient had mildly worsened proteinuria that transiently reached nephrotic range before improving, with normal to low blood pressures. Out of concern for symptomatic hypotension, she was not a candidate for first-line therapy with renin-angiotensin-aldosterone system blockade. In cases of hereditary nephropathies such as X-linked Alport Syndrome, cascade testing may be highly considered to identify family members at risk. Our case demonstrates the variable phenotype of X-linked Alport Syndrome in females and the need for close management during pregnancy, along with the benefits of early genetic testing, given the risks of long-term renal, hearing, and ocular manifestations of this disease.

## Background

Alport syndrome (AS) is a genetic disorder characterized by chronic kidney disease, sensorineural hearing loss, and ocular abnormalities.^1^ It is caused by mutations in the genes encoding the type IV collagen alpha-3, alpha-4, and alpha-5 chains (*COL4A3, COL4A4*, or *COL4A5* genes, respectively). These collagen chains form a heterotrimer that constitutes the basement membrane of the glomerulus, cochlea, cornea, lens capsule, and retina.^1,2^ AS is estimated to occur in 1 in 50,000 live births and affects 30,000–60,000 individuals in the U.S, causing 0.2% of end-stage renal disease (ESRD) cases in adults in the U.S.^1^ Still, the number of affected individuals with AS is likely greater than currently estimated, particularly given its similar presentation with other renal pathologies, such as thin basement membrane nephropathy and focal segmental glomerulosclerosis.^1–3^

AS is inherited in an X-linked (85%) or autosomal recessive (15%) manner, with rare autosomal dominant inheritance.^3^ X-linked Alport syndrome (XLAS) is caused by mutations in the *COL4A5* gene, while autosomal forms are caused by mutations in *COL4A3* or *COL4A4* genes.^1^ Over 2000 mutations in the *COL4A5* gene have been identified in XLAS, consisting of missense (40%) or nonsense (40%) mutations, most commonly with the replacement of a glycine residue. The glomerular basement membrane in particular becomes more prone to proteolytic injury, resulting in glomerular inflammation, tubulointerstitial fibrosis, and progression to ESRD.^1,2^

AS presents similarly in males with XLAS and in males and females with the recessive form, consisting of hematuria and proteinuria that can progress to ESRD. Other symptoms include lenticonus, retinopathy, and retinal thinning.^2^ Typically, X-linked inheritance is suspected when the patient is an affected male, and the disease appears to “skip” a generation. This generational skipping does not signify the absence of disease but rather represents the presence of females with XLAS who remain undiagnosed due to variable phenotypes. Conversely, autosomal recessive inheritance is suspected when the disease only affects one generation or the affected patient is a young female demonstrating more overt symptoms of ESRD, hearing loss, or lenticonus.^2^

Clinical presentation in females with XLAS depends on their mutation type and “lyonization,” the process of random inactivation of one of two X chromosomes in any cell. Lyonization results in a mosaic distribution of the affected collagen in the female kidney and skin, causing variable phenotypes ranging from asymptomatic to severe presentations.^2^ Given this heterogeneity, females with XLAS typically have less severe disease presentations compared to males, yet they are estimated to be affected with the disorder twice as often as men and are often undiagnosed.^2,4^ In addition, female patients with XLAS demonstrate significant renal and hearing/ocular risks. In the European Community AS Concerted Action study, a survey of females with XLAS from 195 families displayed rates of 95% with hematuria, 75% with proteinuria, 28% with hearing loss, and 15% with ocular defects.^5^ Female patients have a lower risk of developing ESRD or deafness before the age of 40 years compared to males, at 10% in women vs 80% in men. However, the risk of ESRD increases after age 60 in females.^2,5^ Given the underdiagnosis and associated renal risks of XLAS, there is a need for further study of the etiology and manifestations of this disorder in female patients.

Here, we present a case of a pregnant woman diagnosed with XLAS caused by a heterozygous mutation in the *COL4A5* gene.

## Case presentation

A 28 year old G1P0 female at 31 weeks and 3 days of gestation was referred from her OB/GYN to our UC Irvine nephrology clinic for proteinuria and hematuria. She had chronic proteinuria, first diagnosed 10 years ago. At the time of referral, the patient had 1+ proteinuria with urine protein/creatinine ratio (UPCR) 0.693 g/g (ref: <0.150 g/g) and urine albumin/creatinine ratio (UACR) 0.308 g/g (ref: <0.029 g/g). She had grossly normal renal function with a baseline serum creatinine of 0.45 mg/dL (ref: 0.57–1.00 mg/dL). She was previously on benazepril for renal protection but stopped the medication when she became pregnant. Systolic blood pressures were in the 110–120 s at home, and she denied any edema. The patient had no prior history of diabetes mellitus, surgeries, smoking, or other comorbidities, and her only medication was prenatal vitamins. The patient had a family history significant for renal pathologies. Her mother and 2 older brothers (ages 35 and 36) had been on hemodialysis for the past 6–8 years, and both brothers also required a renal transplant. However, their exact diagnoses were unknown, and information was limited as her family lived out of the country.

On physical exam, the patient was normotensive with a blood pressure of 101/62. She had a BMI of 23.4 kg/m^2^ (weight 56.1 kg, height 1.55 m). Physical exam was benign with no edema. The patient reported that a prior audiology exam found evidence of minor right hearing loss, but she denied observing hearing deficits in her daily life. In-office exam for sensorineural and conductive hearing loss was normal. She denied any vision difficulties. Initial laboratory work-up showed stable renal function with 1+ proteinuria and mild anemia. UACR was 0.478 g/g, increased from 0.308 g/g 1 month prior. Laboratory results were as follows:

Renal function:

- Serum creatinine 0.47 mg/dL.
- BUN 9 mg/dL.
- eGFR 133 mL/min/1.73 (calculated using the CDK-EPI creatinine equation (2021)).^6^
- BUN/creatinine ratio 19.

Urine studies:

- 1+ blood
- 1+ proteinuria
- UACR 0.478 g/g

Hematologic:

- RBCs 3.39 million cells/μL
- Hemoglobin 10.5 g/dL
- Hematocrit 31.8%
- MCV 94 fl
- Platelets 270 thousand/μL

Serum and urine electrophoresis were negative for gammopathies. In addition, the patient demonstrated high free kappa light chains (24.7 mg/L) and free lambda light chains (28.2 mg/L), but with a normal kappa/lambda ratio of 0.88, suggesting a benign inflammatory response rather than monoclonal gammopathy. She had a negative work-up for autoimmune causes—including systemic lupus erythematosus, Goodpasture’s disease, C3 glomerulonephropathy, and poststreptococcal glomerulonephritis—with a negative work-up for haptoglobin, lactate dehydrogenase, antinuclear antibodies, and anti-DS-DNA antibodies, anti-Smith antibodies, anti-glomerular basement membrane antibodies, complement component 3, complement component 4, complement CH50, anti-DNase B Strep antibodies, and rheumatoid factor. Negative work-up was found for viral hepatitis, HIV, tuberculosis, and syphilis. Hepatic enzymes were normal (AST 16 U/L, ALT 12 U/L). Of note, the patient’s 1,25-dihydroxyvitamin D was elevated at 150 pg/mL, with normal parathyroid hormone (PTH) at 13 pg/mL, but the patient denied any additional supplement use. She ceased her prenatal vitamins following this finding.

Renal ultrasound from a year prior showed a 1.0 cm left renal simple cyst and a 0.2 cm nonobstructing left renal stone, with normal findings of the right kidney. Repeat renal ultrasound showed no evidence of hemodynamically significant renal artery stenosis or signs of cystic disease.

Given the significant family history of renal disease, a genetic component was suspected. Differentials considered included focal segmental glomerulosclerosis, IgA nephropathy, or a congenital origin. Thin basement membrane disease (TBMD) was also considered, given the mild hematuria. AS was deemed less likely given that the patient is female. Genetic testing was ordered with referral to maternal fetal medicine (MFM) to monitor for pre-eclampsia, although the patient was normotensive. MFM recommended induction no later than 39 weeks or sooner if preeclampsia develops.

Genetic analysis revealed a heterozygous c.3587G>A (p.Gly1196Glu) mutation in the *COL4A5* gene, causing XLAS. The variant was predicted to cause a single amino acid missense substitution of glycine to glutamic acid at codon 1196 in exon 40 of the *COL4A5* gene. This variant was located in a highly conserved region of the *COL4A5* protein, predicted to disrupt the Glycine-X-Y motif of the collagen triple helix repeat domain. Computational predictions for the variant predicted a REVEL score of 0.92, with a score above 0.75 strongly suggestive of pathogenicity.^7^ The variant was also absent from the Genome Aggregation Database dataset. Given these findings, the variant was reported as likely pathogenic.

The genetic report also reported the following variants of uncertain significance, all in heterozygous forms: c.841C>T (p.Arg281Trp) of the *PRKCSH* gene, c.4078G>A (p.Glu1360Lys) of the *TSC2* gene, c.333G>A (p.Met111Ile) of the *WDPCP* gene, c.689G>A (p.Arg230His) of the *HRPS1* gene, c.2636C>T (p.Ser879Leu) of the *GRIP1* gene, C.860A>G (p.Gln287Arg) of the *NPHS2* gene, and c.2839C>T (p.Pro947Ser) of the *SLC12A3* gene.

At her 1-month follow-up, the patient’s blood pressure was stable at 118/81. She was due for induction within the next 2 weeks. Repeat laboratory studies showed normal renal function with creatinine 0.49 mg/dL, BUN 9 mg/dL, eGFR 132 mL/min/1.73, and BUN/creatinine ratio 18. Her UACR ratio remained below nephrotic range but had increased to 1.14 g/g, up from 0.478 g/g 1 month prior. She showed continued proteinuria (3+ protein) and hematuria (2+ blood). Her 24-h urine creatinine was normal at 0.99 g/24 h with elevated creatinine clearance of 143 mL/min. Random urine protein was 156.5 mg/dL, and random urine creatinine was 71.6 mg/dL, giving a UPCR of 2.19 g/g. A 24-h urine protein test was not available. Her 1,25-dihydroxyvitamin D remained elevated but decreased from 150 to 132 pg/mL. However, she had mildly low 25-hydroxy-vitamin D at 27.8 ng/mL, along with normal values for PTH of 14 pg/mL, PTH-related-protein <2.0, serum calcium of 8.8 mg/dL, and 24-h urine calcium of 198 mg/24 h. Her blood count showed stable mild normocytic anemia, with RBCs 3.52 million cells/μL, hemoglobin 10.8 g/dL, hematocrit 32.1%, MCV 91 fl, and platelets 300 thous/μL. She had normal liver function with no immediate concern for pre-eclampsia.

The patient was induced 2 weeks later at 38 weeks 5 days and had an uncomplicated vaginal delivery, with a birth weight of 6 lbs, 1 ounce. There were no signs of preeclampsia or other complications. Postpartum urine studies were delayed due to regular postpartum vaginal bleeding. The patient’s son was due to undergo screening by pediatric nephrology.

At follow-up 3 months later, the patient had stable renal function with creatinine 0.52 mg/dL, EGFR 130 mL/min/1.73, and a BUN/creatinine ratio of 25. Urine studies showed UACR 2.25 g/g, increased from 1.14 g/g 3 months prior, but still below the nephrotic range. 24-h urine creatinine was stable at 1.0 g/24 h with creatinine clearance still mildly elevated at 135 mL/min. Her 24-h urine protein was 2.5 g/24 h, with a UPCR of 3.54 g/g, reaching nephrotic range levels for the first time. 25-hydroxy-vitamin D remained stable but mildly low at 27.2 ng/mL. She had a normal blood count with RBCs 4.41 million cells/μL, hemoglobin 12.8 g/dL, hematocrit 39.8%, MCV 90 fl, and platelets 359 thous/μL. While angiotensin converting enzyme inhibitors (ACEi) and angiotensin II receptor blockers (ARBs) were first considered, given their common use in AS, the patient’s systolic blood pressure was routinely in the 90s, and they were thus contraindicated over concerns for hypotension. Spironolactone, a mineralocorticoid receptor antagonist (MRA), was also avoided for risk of hypotension. Sodium-glucose cotransporter 2 inhibitors (SGLT-2i) were further held due to concerns for hypotension and active breastfeeding. We considered but held glucagon-like peptide-1 receptor agonists (GLP-1 RAs) as the patient had a relatively low BMI of 20.78 kg/m^2^ (weight 49.9 kg, height 1.55 m) 1 month postpartum. Given the lack of other options for AS, we decided to practice watchful waiting until an AS-specific drug becomes available through new clinical trials or one of the above medications became appropriate.

**Figure 1.**
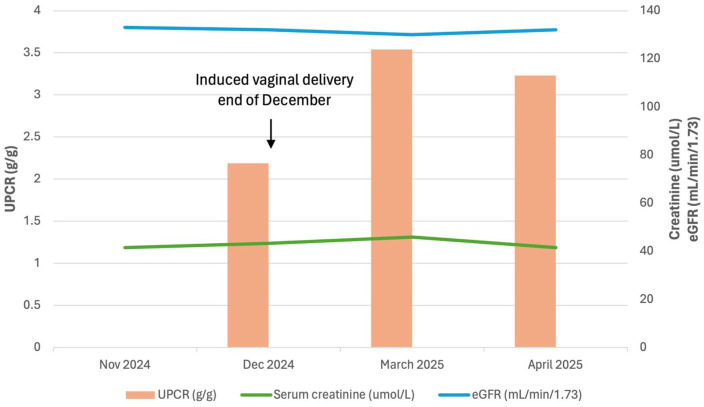
Renal function over time. UPCR: urine protein to creatinine ratio. eGFR: estimated glomerular filtration rate.

At follow-up 1 month later, the patient reported feeling well with systolic blood pressure in the 100–110 s and no dizziness. Renal function was intact with creatinine 0.47 mg/dL, BUN 8 mg/dL, EGFR 132 mL/min/1.73, and BUN/creatinine ratio 17. 25-hydroxy-vitamin D increased to the normal range at 30.9 ng/mL. Her UACR was slightly lower at 2.01 g/g, compared to 2.25 g/g 1 month prior. Urine studies showed 24-h urine creatinine 1.1 g/24 h, creatinine clearance 152 mL/min, and 24-h urine protein of 2.7 mg/24 h. However, her UPCR improved to 3.23 g/g (Figure 1). Her blood count remained within normal limits with no anemia. Renal biopsy was discussed, as it may offer clarity on the type and extent of kidney damage, but the patient opted to continue waiting, which was deemed acceptable given her stable status and confirmed diagnosis. Shortly after the visit, the patient also reported she was being followed by ophthalmology for work-up of a retinal hole. The patient will continue to be monitored with repeat blood work to assess for appropriate interventions, and reports feeling comfortable with this plan. Of note, her son is being followed by pediatric nephrology but is currently asymptomatic and is awaiting genetic testing.

## Discussion

### Clinical presentation

Our study describes a female patient who presented in her 3rd trimester of pregnancy with chronic proteinuria and new-onset hematuria. Genetic testing revealed that our patient had XLAS, with a heterozygous missense mutation in the *COL4A5* gene on genetic analysis. While male patients with XLAS demonstrate classical progressive renal failure, affected females can present with varying severities due to lyonization or random X-linked inactivation.^4,8^

Prior studies of XLAS in pregnancy have shown that non-nephrotic range proteinuria at conception can progress to nephrotic-range proteinuria and fluid overload in the 3rd trimester, even in the setting of normal renal function and arterial blood pressure. Preterm delivery may be necessary due to pre-eclampsia, worsening proteinuria, or unresponsive fluid overload.^9,10^ In our case, the patient demonstrated worsening proteinuria, with an increase in UACR from 0.478 g/g to 1.14 g/g over a 1-month period in the final weeks of her 3rd trimester. However, she did not develop nephrotic ranges of proteinuria, pre-eclampsia, or fluid overload and was able to be induced at term. Post-delivery, the patient’s proteinuria continued to worsen, with UPCR transiently reaching nephrotic range at 3.54 g/g at 3 months postpartum, along with a peak UACR of 2.25 g/g. However, her proteinuria subsequently improved to UACR 2.01 g/g and UPCR 3.23 g/g, respectively. Her proteinuria has otherwise remained subnephrotic throughout her clinical course, with a most recent 24-h urine protein of 2.73 g/24 h. Prior literature shows 15%–30% of women with XLAS developed renal failure by age 60. Our patient’s excursion into nephrotic range proteinuria resolved, but given her young age at presentation, she is still at high risk for renal disease in subsequent years. Her current relatively minor presentation may reflect her specific gene mutation and underscores the heterogeneity of XLAS in females.^2,11^

Of note, our patient demonstrated an elevated 1,25-dihydroxyvitamin D level in her third trimester that lowered slightly after ceasing Vitamin D supplements. Transient increases in 1,25-dihydroxyvitamin D are common physiologic, rather than pathologic, occurrences seen in pregnancy, which is likely the case in our patient.^12,13^ Otherwise, routine measurements of 25-hydroxy-vitamin D levels remained mildly low to normal throughout her course, and she did not have any signs of hypercalcemia or hyperparathyroidism.

### Genotype-phenotype correlation

The patient’s mutation disrupted the conserved Gly-Xaa-Yaa motif of the *COL4A5* protein, which is a common cause of AS, given its resultant aberrant collagen network.^1,2^ This X-linked form aligns with her family history, as her mother was on hemodialysis and her two brothers had required renal transport before age 40, reflective of the more rapid disease progression in males.^1,2^ In cases of inherited nephropathies, particularly in XLAS, where disease presentation can vary, cascade testing may be highly considered to identify family members at risk.^14^

### Differential considerations

TBMD, a type 4 collagen-related nephropathy, is an important differential diagnosis when considering AS, given their similar presentations. TBMD involves the same genes implicated in AS but typically presents with less severe symptoms and rare progression to renal failure, in contrast to our patient’s renal family history.^1^ Therefore, while definitive diagnosis of TBMD requires renal biopsy with electron microscopy, it is less likely in our patient’s case given her family history of renal failure.

On renal biopsy, AS typically presents with initial diffuse thinning of the glomerular basement membrane that can progress to a basket-weave pattern and effaced foot processes with more severe cases.^15^ Our patient declined a renal biopsy at this time; while a biopsy would offer clarity on the extent of any renal disease present, her genetic finding confirms her diagnosis of AS and biopsy was deemed unnecessary given her stable status.^1^

### Treatment constraints

Currently, there is no curative treatment for AS, but supportive treatment generally focuses on limiting proteinuria and renal failure. Prior studies recommend that females with XLAS should be monitored annually for albuminuria and hypertension. Renin-angiotensin-aldosterone-system (RAAS) blockade, in the form of ACEi or ARBs, has been shown to delay the onset of ESRD in XLAS by years, likely through antihypertensive effects, reducing tubulointerstitial fibrosis, and decreasing proteinuria. Thus, RAAS blockade treatment is recommended at the onset of albuminuria, even with normal blood pressure.^2,8,16,17^ Our patient was on the ACEi benazepril prior to pregnancy, but it was stopped given its contraindicated use in pregnancy.^18^ Following delivery, RAAS blockade was held for risk of hypotension given the patient’s baseline blood pressure in the 90s to low 100s. MRA treatments (i.e., spironolactone and finerenone) also pose nephroprotective effects in patients with CKD and DM and have been suggested for AS, but were similarly contraindicated for our patient due to concerns for hypotension.^19,20^

Other potential treatment options include GLP-1 RAs, which have been shown to delay the progression of diabetic CKD, likely through reducing oxidative stress and inflammation and by supporting glomerular basement integrity.^21^ Our patient had a relatively low BMI postpartum (20.78 kg/m^2^), leading us to defer GLP-1RA therapy for risk of undesired weight loss. Additionally, GLP-1RA use is contraindicated during breastfeeding due to a lack of adequate safety data in humans.^22^ Sodium-glucose cotransporter 2 inhibitors (SGLT2i) have also been proposed as treatment options for AS, given their nephroprotective effects in diabetic CKD, and are currently being studied for AS use in the DOUBLE PRO-TECT clinical trial.^23^ However, other than showing benefits in small case studies for AS and immune-mediated glomerular diseases, their efficacy in clinical trial data is limited. SGLTi were held for our patient as they are contraindicated during pregnancy and breastfeeding, along with concerns over volume depletion and hypotension given her low baseline blood pressure.^24–26^

### Emerging therapies

Given the contraindications to available therapies and the patient’s relatively mild presentation, upon shared decision making with the patient, medication changes were deferred for now. However, multiple other AS-specific therapies are under investigation. Hydroxychloroquine, a drug currently used for systemic lupus erythematosus, has shown benefits in reducing hematuria and proteinuria in small samples of patients with XLAS.^27,28^ Other medications currently under review in clinical trials (actively recruiting or active) include endothelin type A receptor inhibitors (i.e., sparsentan, atrasentan) and the small molecule R3R01, which may offer more targeted, immune-mediated treatment options.^27,29–31^

Affected women with XLAS are commonly undiagnosed, but 15%–30% develop renal failure by 60 years and often hearing loss by middle age.^2^ Our patient had evidence of mild right hearing loss on the audiology exam but was otherwise asymptomatic. She also recently began seeing an ophthalmologist for the work-up of a retinal hole, prompting a need for continued follow-up in case of XLAS involvement. She did not have additional ocular findings characteristic of XLAS, such as lenticonus, central/perimacular fleck retinopathy, or temporal retinal thinning.^32^ Limitations of our case include the lack of details on her family history and lack of renal biopsy, which may have offered clarity on her diagnosis; however, her symptoms and genetic mutation strongly support the finding of AS. We will plan to continue monitoring our patient for renal dysfunction and reassess the need for additional therapies.

## Conclusion

Here, we presented a case of a pregnant female patient who was found to have XLAS with a heterozygous mutation in the *COL4A5* gene. The patient demonstrated persistent proteinuria and hematuria throughout her pregnancy course, with no other complications. Pregnancy may exacerbate proteinuria in females affected by heterozygous XLAS, requiring close monitoring to avoid misdiagnosis of pre-eclampsia. In the postpartum period, proteinuria may transiently worsen, as seen in our patient who temporarily reached nephrotic range proteinuria. Our patient’s management was complicated by her baseline low blood pressure, prompting us to clinically defer the primary nephroprotective therapy of RAAS blockade due to the risk of symptomatic hypotension. Our case widens the clinical picture of XLAS in female individuals and demonstrates the variability in presentation. Given the significant renal manifestations of XLAS in females, this study emphasizes the need for early genetic testing and close follow-up in females with suspected type IV collagen disorders.
